# Extrapolating the Coffee and Caffeine (1,3,7-Trimethylxanthine) Effects on Exercise and Metabolism—A Concise Review

**DOI:** 10.3390/nu15245031

**Published:** 2023-12-07

**Authors:** Bernardo Starling-Soares, Marcela Pereira, Guilherme Renke

**Affiliations:** 1 Departamento de Bioquímica e Imunologia, Instituto de Ciências Biológicas, Universidade Federal de Minas Gerais, Belo Horizonte 31310-250, MG, Brazil; 2 Extreme Sports Nutrition Institute—INEE, Belo Horizonte 31310-370, MG, Brazil; 3 Nutrindo Ideais Performance and Nutrition Research Center, Rio de Janeiro 22411-040, RJ, Brazil; 4 Clementino Fraga Filho University Hospital, Federal University of Rio de Janeiro, Rio de Janeiro 21941-901, RJ, Brazil

**Keywords:** caffeine, insulin resistance, obesity, exercise, thermogenic effect

## Abstract

The consumption of coffee and caffeine (1,3,7-trimethylxanthine) is part of many cultures worldwide. Their properties include serving as a neurostimulant aid, enhancing energy substrate levels, and improving general exercise performance. Both present therapeutic effects that can also be used to control chronic and metabolic diseases due to four mechanisms: adenosine receptor antagonism, increased catecholamine concentrations, a phosphodiesterase inhibitor, and a stimulator of calcium-release channels. Despite the individual genetic variabilities, distinct mechanisms have been demonstrated to improve physical performance, thermogenesis, lipolysis, insulin sensitivity, and hormonal modulation. Thus, coffee consumption and caffeine supplementation may enhance physical and mental performance and may improve metabolic variables, reducing oxidative stress, inflammation, and insulin resistance. Current data reveal vital aspects of coffee and caffeine consumption in specific populations, although further studies are needed to define clinical interventions with caffeine in obesity and chronic conditions.

## 1. Introduction

The primary and most widespread form of caffeine consumption is through coffee beverages, estimated to reach an impressive 2.25 billion cups consumed worldwide daily—approximately 530 million liters/day [1]. It is believed that the consumption of coffee originated in the northeastern region of Africa and gradually spread across the Middle East during the 15th century, eventually making its way to Europe [2]. The coffee beverage has a highly complex composition, comprising numerous chemical constituents, often reaching several hundred in number. The volatile fraction of coffee has been extensively studied, accounting for most of the identified components within the beverage [3]. Beyond coffee, caffeine is present in various beverages such as soft drinks, tea, cola nuts, and cocoa, as well as in supplements, other foods (ordinarily in trivial concentrations), and numerous pharmaceutical preparations, such as muscle relaxants, decongestants, and allergy drugs [4]. Its properties include serving as a neurostimulant aid, enhancing energy substrate levels, and improving general exercise performance [5]. Caffeine (1,3,7-trimethylxanthine), after ingestion, is demethylated into three pharmacologically active dimethylxanthines: theophylline, theobromine, and paraxanthine—all of them with similar molecular adenosine structure—with paraxanthine being the primary metabolite in blood after caffeine absorption [2].

## 2. Coffee and Caffeine Outcomes

### 2.1. Caffeine Action Mechanisms 

**Figure 1:**
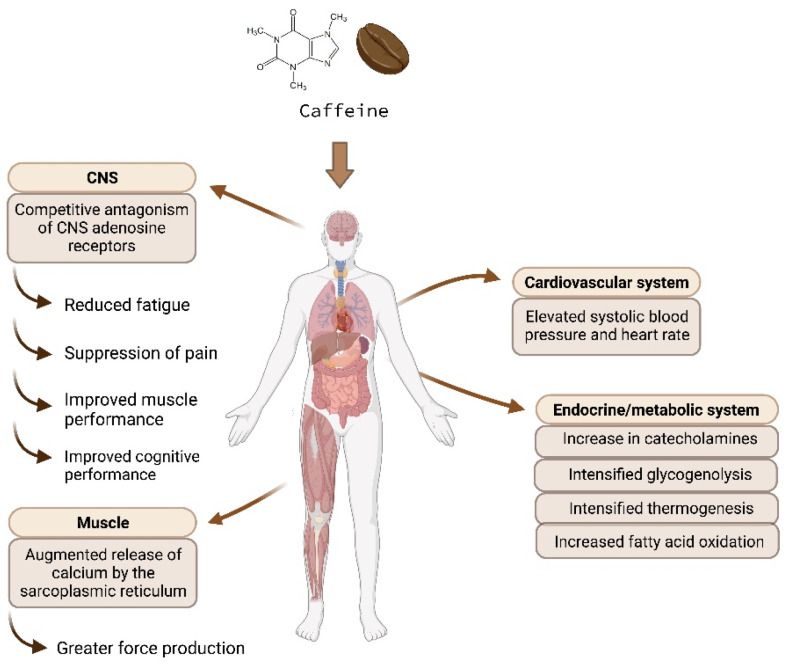
(Created with BioRender). Action mechanisms of caffeine (CNS: central nervous system).

Caffeine exerts its effects through four described mechanisms (Table 1 and Figure 1): adenosine receptor antagonism (1) and increased catecholamine concentrations (2). At plasma concentrations of 5–10 μmol/L, caffeine partially blocks the function of adenosine receptors (1), which contributes to its stimulant properties. Additionally, caffeine crosses the blood–brain barrier and raises circulating concentrations of epinephrine/adrenaline (2). The proposed mechanisms of caffeine as a phosphodiesterase inhibitor (3) or a stimulator of calcium-release channels (4) are considered less significant, as these actions require higher concentrations of caffeine, typically in the range of 500–5000 μmol/L, which are significantly higher than the concentrations achieved through regular coffee consumption (representing 5–20 μmol caffeine/day) [2]. Furthermore, caffeine is considered a weak phosphodiesterase inhibitor, according to studies conducted by Daly and Fredholm [6]. In summary, caffeine’s primary mechanisms of action involve blocking adenosine receptors and increasing catecholamine levels. These mechanisms contribute to the **acute effects** of caffeine, such as increased energy expenditure, heart rate, lipolysis and fatty acid oxidation rate, hyperglycemia, and hyperinsulinemia. Caffeine/coffee also has **chronic effects**, including weight loss, body fat loss, muscle hypertrophy, and improved insulin sensitivity, similar to other β-agonists like ephedrine [7,8].

### 2.2. Caffeine Metabolism and Pharmacokinetics Differences between Populations

Even before Sachse and colleagues described in their seminal work (1999) the different individual slow (AC and CA) and fast (AA) metabolizer genotypes (163C>A; rs762551) of cytochrome P450 *CYP1A2* (Cytochrome P450 Family 1 Subfamily A Member 2) [15], studies presenting different pharmacokinetics and physiologic actions of caffeine between eutrophic, postobese and obese subjects (exercising or not) had been topics of investigation [16]. In an interesting and previous study by the Kamimori laboratory, significant findings regarding the impact of caffeine on the response of free fatty acids in obese individuals during periods of rest were demonstrated. The results presented a noteworthy delay in the free fatty acid response when caffeine is administered to obese individuals compared with eutrophic subjects. This indicates that the metabolic effects of caffeine on obese individuals exhibit distinct characteristics compared to those observed in non-obese individuals. Untrained and caffeine “naïve” subjects received oral caffeine (5.83 mg·kg^−1^ lean body weight) or placebo (50 mg citrate) in two situations: before 3 h of seated rest and before a 90 min of treadmill walking (40% of their maximal aerobic power) succeeded by 90 min of seated recovery. Examining the pharmacokinetic parameters of caffeine in obese individuals at rest yielded distinct differences compared to their lean counterparts. Specifically, obese individuals exhibited a higher absorption rate constant (Ka), a lower elimination rate constant (Ke), and a longer half-life (t^1/2^) of caffeine. Substantial difference observed in the volume of distribution (Vz) between the obese and lean groups at rest was found: 91% greater (103.1 vs. 54.1, respectively). The findings revealed a consistent pattern when examining the impact of exercise on the pharmacokinetics of caffeine in obese individuals. Specifically, exercise consistently reduced both the maximal serum concentration of caffeine and the area under the curve, indicating a lower overall exposure to caffeine in the serum—effects that were not consistently observed in lean individuals. These results demonstrate that obesity, as well as exercise, can alter the normal pharmacokinetics of caffeine. In simpler terms, when someone is at rest, there may be a difference in the distribution of caffeine in their body due to its highly fat-soluble nature. Caffeine can enter fat cells (adipocytes) due to its postabsorption high concentration in the bloodstream and stay in the adipocytes for some time. As the caffeine levels in the bloodstream decrease over time, the caffeine stored in fat cells will diffuse back into the bloodstream and eventually reach the liver. This process is more significant in obese individuals because they have more fat tissue, which means a larger amount of caffeine can be stored in their bodies. As a result, the caffeine remains in their bloodstream for a longer period compared to non-obese individuals [16]. 

*CYP1A2*, as already mentioned, and *ADORA2A*—the gene encoding adenosine receptor A2A (polymorphism at 1976C>T; rs5751876)—are pivotal genes influencing the ergogenic effects of caffeine, with *CYP1A2* primarily responsible for caffeine and caffeine derivatives’ metabolism and *ADORA2A* related to caffeine-induced anxiety effects. *ADORA2A* genotypes have a frequency of approximately 45% C/T allele carriers and T/T and C/C carriers ranging between 20–30%. These genetic factors may contribute significantly to the observed interindividual variations in responses to caffeine intake [17]. 

Approximately 40% of the general population carries the A/A *CYP1A2* genotype, while 50% and 10% have the A/C and C/C *CYP1A2* genotypes, respectively. Studies have reported varying performance outcomes associated with these genotypes. Some research demonstrated more significant performance improvements in individuals with the A/A genotypes, while others found no significant differences or improved performance in individuals with the A/C genotype. Notably, these studies examining the impact of *CYP1A2* genotype on caffeine ergogenicity, showing inconsistent results, have possibly been influenced by the variety of the protocols established. It is essential to acknowledge that the effectiveness of caffeine as an ergogenic aid is most pronounced in endurance exercises, and the choice of a shorter-duration protocol may compromise the reliability of study outcomes [17]. 

More recently, Guest and colleagues identified that polymorphism in *HTR2A* (serotonin receptor type 2 gene) (polymorphism at 102C>T; rs6313)—which influences serotonin receptor signaling activity—has been implicated in the modulating effects of caffeine action on exercise performance. *HTR2A* genotypes present a frequency in this study of 48% C/T allele carriers and T/T and C/C carriers 52%. The findings indicate that 4 mg caffeine per kg of body mass improves performance in individuals with the HTR2A CC genotype but specifically in subjects who are also CYP1A2 fast metabolizers [18]. 

Interestingly, people with genetic polymorphism linked to slower caffeine processing tend to drink smaller quantities of coffee, even presenting elevated blood caffeine levels [17,19]. A link between higher predicted blood caffeine levels (genetically derived) and a decreased risk of type 2 diabetes is partially attributed to reduced total body fat, a known significant factor in the disease [19]. 

### 2.3. Coffee x Caffeine Effects on Physical Performance 

Athletes, known for their readiness to adopt performance-enhancing practices, have been aware of the advantageous effects of caffeine for years (Table 2). Aguilar-Navarro and coworkers reported that approximately 75% of athletes consume caffeine near competitive events, recognizing its ergogenic benefits [20]. As many different forms can be used to attain the performance-enhancing benefit, studies in the 90s started to test if coffee beverages—as the most daily common form of caffeine use—could bring the same effects as caffeine capsules. Graham, Hibbert, and Sathasivam stated, “Caffeine ingestion increases plasma epinephrine and exercise endurance… and these results have been frequently transferred to coffee consumption” [21]. Aiming to understand better why original research by Costill and coworkers [22] that measures the endurance enhancement effect of coffee beverage pre-exercise presented relatively minor improvements (21%) than those seen in their test of the same characteristics (43% in some cases, but using caffeine capsules), they designed a new study, where subjects ingested the following five protocols: placebo capsules (dextrose) and water, caffeine capsules and water, regular coffee, decaffeinated coffee, or decaffeinated coffee plus caffeine. The ingestion of caffeine in capsules resulted in a higher endurance time of 9.9 min or 31% over placebo capsules and 7.6 min or 22.8% over that for decaffeinated coffee. All protocols used 4.45 mg caffeine/kg [21]. 

Interestingly, caffeine from coffee resulted in the same plasmatic caffeine concentration, but the epinephrine/adrenaline response was just 50% of that seen with the capsules. The group concludes and proposes that these results suggest that “cholinomimetic factor(s)” present in coffee may suppress some sympathetic responses in both central and adrenal systems [21]. However, the study is not free from issues: 1—the number of subjects was low (n = 9), and as it is known to exist genetically different types of metabolizers (slow and fast genotypes) [15] the sample was probably too low to prove their results; 2—although they proposed a moderate dose use (4.45 mg caffeine/kg) in the purpose section, when they presented the significant difference seen from Costill and their previous tests [21], they cited that the dose used was a high dose of caffeine (9 mg/kg); 3—90% of the participants correctly guessed the placebo capsules, and 80% identified the caffeine capsules [22]. 

Previously, Tse isolated a compound with cholinergic action derived from regular and decaffeinated coffees, and after purifying it, demonstrated that injecting it in animals (rats) resulted in declines in heart rate and blood pressure. Interestingly, this compound is found only in roasted coffees and not in green coffee beans, which is probably a product-derived browning reaction that occurs during roasting [3]. Other publications related to the theme present other important substances in coffee (such as chlorogenic acid and quinidine and their actions on glucose metabolism and antioxidant effects) [2]. Despite the tremendous commercial popularity, none of the publications used green coffee as a group intervention protocol for comparing exercise performance enhancement. Its use could be an innovative form to study the actions of different derived caffeine forms, as it is known that food and its ingredients influence the absorption and kinetics of methylxanthines [23].

**Table 2:** **Effects of caffeine supplementation on physical performance** (AUC: area under the curve; g: grams; h: hour; kg: kilograms; km: kilometers; mg: milligrams; POMS: profile of mood states; U.S.: United States).

Author(s), Year, Reference Number	Caffeine Dose	Timing	Characteristic of Study/Participants	Results
Lieberman et al., 2002 [12]	Caffeine in doses of 100, 200, or 300 mg.	The experiment commenced on Sunday night, and at the following Wednesday night at 9:30, either caffeine or placebo was administered.	Double-blind, placebo-controlled study. A total of 68 U.S. Navy Sea–Air–Land trainees, randomly allocated to receive either 100, 200, or 300 mg caffeine or placebo capsules, after 72 h of sleep deprivation and sustained exposure to other stressors.	Caffeine (200 and 300 mg) yielded statistically significant enhancements in visual vigilance, choice reaction time, repeated acquisition, self-reported fatigue, and sleepiness. Most pronounced effects were observed in assessments related to vigilance, reaction time, and overall alertness.
Ruiz-Moreno et al., 2020 [24]	3 mg of caffeine per kg of body mass.	60 min before the onset of exercise.	Randomized double-blind, placebo-controlled study; 12 healthy participants undertook two acute experimental trials after ingesting of caffeine (3 mg/kg) or a placebo. Trials consisted of 1 h of uninterrupted cycling at Fatmax. Energy expenditure, fat oxidation rate, and carbohydrate oxidation rate were continuously evaluated by indirect calorimetry.	Caffeine augmented the total fat oxidized during the trial and reduced the amount of carbohydrate oxidized and the mean self-perception of fatigue in comparison to the placebo.
San Juan et al., 2019 [25]	6 mg of caffeine per kg of body mass.	75 min before the start of the session.	Randomized double-blind, placebo-controlled study evaluated the effects of caffeine supplementation on anaerobic performance, neuromuscular efficiency and fatigue in upper and lower extremities in 8 Olympic-level boxers. The athletes performed 2 test bouts after the ingestion of caffeine or placebo.	Caffeine supplementation improved anaerobic performance without affecting electromiography activity and fatigue levels in the lower limbs. Other observed benefits were improved neuromuscular efficiency in some muscles and reaction speed.
Foskett et al., 2009 [26]	6 mg of caffeine per kg of body mass.	60 min before exercise.	Randomized double-blind, placebo-controlled study. A total of 12 male soccer athletes participated in two 90 min sessions of soccer-specific intermittent running, with intervals dedicated to assessments of their soccer skill.	Participants incurred a significantly reduced amount of penalty time (added for erros) in the caffeine trial, resulting in a notably diminished total duration of this particular trial. The ingestion of caffeine led to enhancements in players’ passing accuracy and jump performance, with no adverse impact observed on other performance indicators.
Jodra et al., 2020 [27]	6 mg of caffeine per kg of body mass.	60 min before exercise.	Randomized double-blind, placebo-controlled. 8 elite athletes (senior boxing national team) were examined alongside 10 trained recreational athletes the immediate effects of caffeine on anaerobic performance, mood, and perceived effort in both group. During two separate experimental conditions, involving either caffeine supplementation or a placebo, the athletes underwent a Wingate test.	Caffeine supplementation improved anaerobic performance in both groups. The ergogenic effect of caffeine on several mood dimensions and subjective evaluation was superior in the elite athletes.
Domínguez et al., 2021 [28]	6 mg of caffeine per kg of body mass.	75 min before exercise.	Randomized double-blind, placebo-controlled study examined 15 male resistance training experience volunteers. A total of 60 min after supplement ingestion, participants filled two questionnaires, measuring subjective vitality and mood state. Participants’ performance was evaluated through a Wingate test, which was followed by measurements of rate of general perceived exertion, muscular and cardiovascular level.	Caffeine supplementation improved some components of mood assessed by POMS (tension and vigor dimensions) and subjective vitality profiles, which were succeeded by a enhanced maximum power, average power, and lower time needed to reach maximum power during the Wingate test. Caffeine supplementation yields positive effects both in psychological and physical aspects in trained volunteers.
Clarke et al., 2019 [29]	0.09g of coffee per kg providing 3 mg of caffeine per kg of body mass.	60 min before exercise.	Randomized double-blind, placebo-controlled study. 38 recreationally active participants executed a 5 km cycling time trial on a cycle ergometer after ingestion of 0.09 g·kg^−1^ coffee, a placebo or control (water). Coffee intake significantly augmented salivary caffeine levels.	Coffee ingestion achieving 3 mg·kg^−1^ of caffeine, increased salivary caffeine levels and augmented 5 km cycling time trial performance in men and women by a similar degree (lowering by approximately 9 s and 6 s following coffee ingestion compared with placebo and control, respectively).
Loureiro et al., 2021 [30]	The total caffeine provided with the three doses of coffee + milk treatment was 8 mg per kg of body mass.	Three doses of coffee + milk at times 0, 60 and 120 min after exercise (recovery time).	Randomized double-blind, placebo-controlled study. 14 endurance-trained men executed an exhaustive cycle ergometer exercise depleting muscle glycogen. In the following morning, subjects performed a second cycling protocol followed by a 4-h recovery, whilst they received either test beverage (coffee + milk) or control (milk) and a breakfast meal, in a simple randomization model. Blood samples and muscle biopsies were realized at the onset and by the end of recovery.	The ingestion of coffee + milk resulted in greater muscle glycogen recovery, greater glucose and insulin total AUC compared with just milk. The inclusion of coffee to a beverage with adequate quantities of carbohydrates augmented muscular glycogen resynthesis, the glycemic and insulinemic response during the 4 h recovery after exhaustive cycling exercise.

Liguori and colleagues (1997) intending to report a statistical comparison of the time to peak and peak level between coffee, cola and capsules of caffeine as vehicle, presented that although mean peak saliva caffeine levels were not distinct between coffee and cola (42 to 39 min and 9.7 to 9.8 micrograms/mL, respectively) salivary caffeine concentration was faster and higher following coffee ingestion compared with the caffeine capsule (42 to 67 min and 9.7 to 7.8 micrograms/mL, respectively) for the same 400 mg dose intake. This timing factor can be an important point when defining the studies’ protocols wanting to guarantee more reliable results, as the specific time when the peak time is achieved in the distinct activities can be a differential factor for performance [31]. Hodgson and colleagues (2013), still wanting to clarify the coffee x caffeine effects, investigated the ergogenic effects of anhydrous caffeine in liquid form (99.8% pure, dissolved in 600 mL of water) to coffee (instant coffee in 600 mL of water) and placebos (decaffeinated coffee in 600 mL of water or 600 mL of water with quinine, to mimic the bitter coffee taste) ingestion in a cycling time trial test. Using eight trained male cyclists in a crossover study model, they observed that both coffee and caffeine trials were beneficial to exercise performance by similar magnitudes (5.0% faster than placebos) [32]. Trexler and colleagues (2016) reported that coffee intake (8.9 g of dehydrated coffee in 350 mL of water, yielding 303 mg caffeine, 30 min before testing) is a suitable source of caffeine to improve repeated sprint performance—especially at weeks of multiple strenuous bouts—but not in the strength exercises of fifty-four resistance-trained males [33]. Distinctly, Richardson and Clarke (2016) concluded that nine resistance-trained males during resistance exercise lifted more total weight during back squats following coffee ingestion (0.15 g/kg body weight of caffeinated coffee in 600 mL of water), when compared with a placebo [34]. 

### 2.4. Caffeine Thermogenic and Insulin Sensitivity Effect (Acute x Chronic)

Greenberg, Boozer, and Geliebter searched for epidemiologic published studies relating associations between habitual coffee consumption, diabetes risk, and glucose metabolism effects. None of the studies found a deleterious effect, and just 3 out of 20 did not find any protective effect [2].

There is a notable difference between clinical trials evaluating the acute effect of coffee and trials evaluating its chronic impact—usually lasting longer than 4 weeks [35]. Acute doses of caffeine can impair insulin sensitivity and glucose tolerance through the antagonism of the A1 and A2 adenosine receptors, thus altering the glucose uptake in skeletal muscles—or by crossing the blood–brain barrier and stimulating epinephrine release: an indirect/systemic effect [36,37]. Lee and colleagues, investigating the effect of caffeine ingestion on insulin sensitivity, found that ingestion of a single dose of 5 mg/kg body weight of caffeine can impair insulin effects by a similar degree in the lean (33%), obese (33%), and type 2 diabetic (37%) subjects tested by a hyperinsulinemic–euglycemic clamp procedure. Even after a prescribed exercise protocol, the caffeine dose ingestion was still associated with a significant decrease in insulin sensitivity (*p* < 0.05) [38], showing that in short-term (acute) trials, the hyperglycemic effects of caffeine are dominant over the possible beneficial effects of the other constituents of the coffee.

On the other hand, coffee contains a rich source of other phytochemicals such as chlorogenic acid (CGA) and trigonelline, which may improve glucose metabolism through beneficial effects on oxidative stress, proinflammatory state, gluconeogenesis, insulin secretion, glucose uptake, and intestinal glucose absorption [37]. It has been estimated that chronic consumption of six cups of coffee (i.e., 600 mg caffeine/day) causes an increase in energy expenditure of approx. 100 kcal/d by its thermogenic effect [4]. The initial and seminal investigation into the impact of caffeine on thermogenesis can be traced back to the work conducted by Hoppe in 1857. Notably, Hoppe observed a certain degree of elevation in carbon dioxide output after caffeine administration [39]. Subsequently, Edward Smith, two years later, undertook a comprehensive examination of various facets of respiration, encompassing the effects of substances such as tea and coffee. Smith’s investigation was executed with great expertise and proficiency, contributing significantly to understanding the subject matter [40]. 

One study that assessed the effect of weight change found a prospective protective effect of beverage consumption that occurred only in participants who had previously lost weight [41]. The protective effect occurred after weight loss and was related to weight loss in a dose response [2].

Several studies, both conducted in animals and humans, have shown that long-term coffee consumption has anti-inflammatory solid properties from coffee components (caffeine, CGA, cafestol, trigonelline, kahweol, caffeic acid, and ferulic acid) [37,42]. Oxidative stress and progressive inflammation are often paralleled and are considered pathological in developing type 2 diabetes and associated complications [43]. Clinical studies have shown that coffee consumption decreases inflammatory markers, such as IL-1β, IL-6, TNF-α, C-reactive protein, monocyte chemotactic protein 1, vascular cell adhesion molecule 1, C-peptides, endothelial-leukocyte adhesion molecule 1, and IL-18 [35].

In type 2 diabetes, glucolipotoxicity can trigger inflammation by initiating a proinflammatory signaling cascade, resulting in the translocation of the nuclear factor NF-kB. This, in turn, activates a range of proinflammatory indicators. CGA found in coffee could help thwart the onset of these inflammatory markers by inhibiting the action of NF-kB. [37]. Past studies have shown that CGA can effectively suppress the initiation cascade of NF-kB in cell cultures [44]. 

A cross-sectional study of healthy women and women with type 2 diabetes from the Nurses’ Health Study I cohort assesses the relationship between long-term coffee consumption and inflammation markers and endothelial dysfunction markers. The consumption was associated with lower plasma concentrations of E-selectin and C-reactive protein in women with type 2 diabetes, features typically elevated in inflammatory states [45]. 

A randomized parallel-arm intervention was conducted with 45 healthy, overweight volunteers who were regular coffee consumers. In this 8-week randomized trial, the level of adiponectin increased for the caffeinated coffee group compared to the group receiving no coffee. The protein fetuin-A levels decreased for the decaffeinated coffee group compared with no coffee at the end of the intervention. Improvements in adipocyte and liver function, as indicated by changes in adiponectin and fetuin-A concentrations, may contribute to long-term coffee consumption’s beneficial metabolic effects [46]. 

A recent review that discusses the association between chronic coffee consumption and a lower risk of type 2 diabetes finds that ingesting coffee phytochemicals induces an adaptive cellular response characterized by upregulation and de novo synthesis of enzymes involved in cell defense and repair. A key regulator is the nuclear erythroid 2-related factor 2 (Nrf2) associated with the aryl hydrocarbon receptor, AMP-activated kinase, and sirtuins. The available data suggest that a Nrf2-dependent mechanism for the antidiabetic action of coffee may act on the beta pancreatic cell. Activation of Nrf2 leads to sustained upregulation of antioxidative defense, improved mitochondrial function and biogenesis, and prevention of cell damage during periods of high insulin secretion in the prediabetic state [47].

## 3. Discussion

The prevalence of metabolic diseases such as type 2 diabetes is increasing worldwide, with the number of affected adults nearly quadrupling in the last 40 years [48], while the pathological features of obesity, insulin resistance, oxidative stress, and inflammation are increasing predominantly implicated in the development and acceleration of cardiovascular diseases [49,50]. Regarding this, coffee consumption, caffeine supplementation, and other natural adjuvant compounds are promising for several treatments of metabolic disorders. Chronic coffee-related mechanisms, such as caffeine’s thermogenic and metabolic-helping effects, antioxidant and anti-inflammatory effects, and improved mitochondrial metabolism, may help treat these conditions.

Furthermore, as demonstrated in this review, coffee’s bioactive compounds are also widely explored for their ability to improve exercise capacity and performance in athletes. It has been shown that low-to-moderate doses (2–6 mg/kg) of caffeine are necessary to improve aerobic performance and muscular strength, with a benefit similar in magnitude within this dosage. Doses greater than 6 mg/kg are also ergogenic, but the prevalence of side effects usually increases with the dose [5,51,52].

As previously demonstrated, in addition to improved physical performance, current data indicate that coffee-caffeine consumption, potentially due to its abundant antioxidants, is associated with improved oxidative stress and insulin resistance [53].

Additionally, caffeine counteracts the harmful effects of oxidative stress and inflammation, potentially reducing the increased risk of metabolic diseases [53,54]. This is consistent with preclinical evidence [55,56] on the proposed mechanisms by which coffee helps the toxic effects of inflammation and oxidative stress in various tissues to increase its potential benefits. The primary mechanism explaining the enhanced therapeutic effects of coffee or its bioactive compounds is the increase in intracellular antioxidants through the activation of nuclear factor erythroid 2-related factor 2 (Nrf2) [57,58,59].

Synergistically, caffeine influences energy balance by increasing energy expenditure and decreasing energy intake; therefore, it can potentially be considered a body weight regulator in obesity and these metabolic disorders. Caffeine can improve energy balance when consumed in low-to-moderate doses (2–6 mg/kg) by physically active or sedentary individuals (obese or normal-weight men and women) [60]. Caffeine is associated with increased energy expenditure; thus, caffeine counteracts the decrease in basal metabolic rate that commonly occurs during periods of weight reduction and restrictive diets. Furthermore, obese individuals with reduced thermogenesis may have difficulty losing weight with conventional treatment. Even with more significant accumulation and response to caffeine, treating these patients is challenging in clinical practice. Therefore, in obese people who could require a low-calorie diet, possibly also controlled by drug treatment, it would be reasonable to use caffeine as a metabolic activator. A therapy that combines caffeine with a calorie-shifting diet could be an exciting alternative for weight and fat loss, with slight changes in resting metabolic rate and better tolerance of individuals to the new diet [60,61].

Regular coffee consumption has been associated with a low risk of obesity, metabolic syndrome, and TDM2. Caffeine can be used as a weight control regulator and prevention of comorbidities. As caffeine consumption increases, the risk of obesity and diabetes decreases [62]. This opens the way for more research to examine how coffee or its active ingredients modulate intracellular mechanisms related to energy metabolism, hormone secretion, lipid metabolism, glucose uptake, etc. and even in other pathology conditions as it has demonstrated to be helpful in reducing the risk of cardiovascular diseases, the risk of gallstone formation, the risk of Parkinson’s disease development, the risk of depression and suicide, and the risk of cognitive decline, as well as being an adjuvant for headache treatment [63,64,65,66,67,68] (Table 3).

In the experience of our medical treatment center, Nutrindo Ideais, specialized in obesity and sports performance, genetic variability and the caffeine administration route can make a total difference in the patient’s clinical response and treatment follow-up. In our expertise, using oral caffeine in tablets or capsules with dosages varying between 100 and 500 mg, the results engage in some subgroups. We also observed good safety in using benzoic caffeine as an intramuscular injection with dosages changing between 100 and 200 mg, with a frequency of weekly or every three days. Furthermore, regarding sports performance, we emphasize that we must be careful with interpreting results previously observed in the literature due to the heterogenicity of most studies. We cannot extrapolate the results found in high-performance athletes to amateur athletes and patients with chronic clinical conditions.

Furthermore, we advise on the adverse effects of a high intake or use of caffeine on health, especially in recreational athletes or patients with chronic diseases. Exaggerated consumption of coffee and caffeine can excessively influence the cardiovascular system through their positive inotropic and chronotropic effects, which can be harmful to health. Additionally, due to the activating effects of the central nervous system, some people may develop an addiction syndrome. This is because caffeine improves memory concentration and improves physical performance. Some individuals develop an uncontrolled need to consume caffeine, which can characterize dependence or abuse when withdrawal and tolerance mechanisms develop chronically [69,70].

The rate of adverse events may increase due to risk factors, especially cardiovascular events in individuals who consume caffeine from other sources (e.g., energy drinks). Excessive use can lead to caffeine overdose, as has been reported in the literature [69]. A review by Nawrot et al. [71] shows that the maximum safe dose of caffeine is 400 mg/day (approximately 6 mg/kg). However, acute caffeine ingestion with doses above 3 g can cause significant side effects, with the occurrence of arrhythmia being more common, which can even be fatal, as already observed [69].

Although controversial, we emphasize that this risk and sensitivity to caffeine may be even more significant in individuals with some genetic polymorphisms. Despite this, further studies are still needed to expand our understanding of gene variability in caffeine metabolism. Thus, understanding the genetic differences in caffeine metabolization and pharmacokinetics between populations poses a challenge for further investigations. Different doses of caffeine should be tested, especially comparing men and women, overweight individuals, and metabolic diseases, to determine its potential therapeutic benefits that improve human health.

## 4. Conclusions

Caffeine consumption, whether in coffee or as a supplement, has described benefits in metabolism, improved physical performance, cognition, thermogenesis, insulin sensitivity, and hormonal modulation. Its use is safe in low-to-moderate doses (2–6 mg/kg) with apparently dose-dependent improvements. However, genetic variability and the need for more standardization of doses may generate divergent results in the literature and may not reflect clinical practice. This review summarizes the effects of caffeine on physical performance and as a potential therapeutic option for metabolic conditions, especially type 2 diabetes and overweight. New studies are needed to define supplementation protocols and dosages in metabolic diseases. Evidence remains lacking, especially in women and patients with chronic conditions. 

## Figures and Tables

**Table 1:** Action mechanisms of caffeine (CNS: central nervous system).

Action Mechanisms of Caffeine	References
Increased fatty acid oxidation rate.	Varillas-Delgado et al., 2023 [8]
Competitive antagonism of CNS adenosine receptors, resulting in diminished tiredness, pain suppression, and enhanced muscular function.	Davis et al., 2003 [9]
Elevated systolic blood pressure and heart rate by blocking adenosine receptors.	Casiglia et al., 1991 [10]
Greater catecholamines levels lead to CNS stimulation and intensified glycogenolysis.	Stuart et al., 2005 [11]
Improved cognitive performance.	Lieberman et al., 2002 [12]
Augmented release of calcium by the sarcoplasmic reticulum and greater force production.	Weber and Herz, 1968 [13]
Thermogenesis and higher energy expenditure.	Clark, 2019 [14]

**Table 3:** Benefits of caffeine ingestion for other disorders.

Reduced risk of cardiovascular diseases	An inverse association has been observed between coffee consumption and coronary artery disease, stroke, and death from cardiovascular causes.	Ding et al., 2014 [63]
Reduced risk of gallstones	Coffee consumption has been associated with a reduced risk of symptomatic gallstone disease.	Nordestgaard et al., 2019 [64]
Reduced risk of Parkinson’s disease	There is a decreased Parkinson’s disease risk with caffeine consumption.	Qi and Li, 2014 [65]
Reduced risk of depression and suicide	Prospective studies support an association between caffeine consumption and lower risk of suicide.	Lucas et al., 2014 [66]
Reduced risk of cognitive decline	Higher coffee consumption was associated with slower cognitive decline in cognitively normal elderly.	Gardener et al., 2021 [67]
Headache	Caffeine is an adjuvant for the acute treatment of headache in combination with analgesics.	Lipton et al., 2017 [68]

## Data Availability

There are no novel data associated with this review article.
